# Supplementary material for: Mycotransformation of Commercial Grade Cypermethrin Dispersion by Aspergillus terreus PDB-B Strain Isolated from Lake Sediments of Kulamangalam, Madurai
Source: Molecules. 2024 Mar 23;29(7):1446. doi: 10.3390/molecules29071446 (PMC11012587; doi:10.3390/molecules29071446)
Supplement: Supplementary file 1 [file molecules-29-01446-s001.zip › molecules-2856812-supplementary.pdf]

Supplementary Table S1. Physicochemical parameters of the lake sediment samples used in the study

Parameter	Unit	Value	Interpretation
**Texture**	-	-	clay loam
**Calcareousness**	-	-	Very high
**pH**		7.35	neutral
**EC**	dS/m	1.87	Critical for germination
**Available nitrogen**	Kg/ha	106	low
**Available phosphorus**	Kg/ha	59	High
**Available potassium**	Kg/ha	312	High
**Organic carbon**	g/Kg	6.46	Medium
**Available iron**	mg/L	12.10	Sufficient
**Available manganese**	mg/L	16.67	Sufficient
**Available zinc**	mg/L	0.85	deficient
**Available copper**	mg/L	4.18	sufficient
**Total nitrogen**	%	0.11	-

Supplementary Table S3. Screening results of the fungal strains for the production of different enzymes

Fungal strain	Protease	Caseinase	Cellulose	Urease	Esterase	β-glucosidase
**A**	-	-	-	-	-	-
**B**	-	-	-	-	-	+
**C**	-	-	-	-	-	-
**D**	-	-	-	-	-	-
**H1**	-	-	-	-	-	-
**H2**	-	-	-	-	-	-
**H3**	-	-	-	-	-	+
**I**	-	-	-	-	+	+
**J**	-	-	-	-	-	-
**UN 2**	-	-	-	-	-	-
**UN 3**	-	-	-	-	-	+
**S+M 108**	-	-	-	-	-	-
**M3 101**	-	-	-	-	-	+
**M3 106**	-	-	-	+	-	-
**M3 107**	-	-	-	-	-	-
**M3 108**	-	-	-	+	-	+

Supplementary Table S4. Screening results for the production of lignin-degrading enzymes

Fungal strain	Bromophenol blue	Methylene blue	Phenol red	Staining lignin	Congo red	Guaiacol	Tannic acid
**A**	-	-	-	+	+	-	+
**B**	-	+	-	+	-	-	+
**D**	+	-	-	+	-	-	+
**H**	+	+	-	+	-	-	-
**I**	+	-	+	+	+	-	+
**J**	+	-	-	+	+	-	-
**UN2**	-	-	+	+	+	-	+
**UN3**	+	-	-	+	+	-	-
**S+M10^{8}**	+	-	-	+	+	-	+
**M3 10^{1}**	-	+	+	+	-	-	+
**M3 10^{6}**	-	-	-	+	-	+	+
**M3 10^{7}**	-	-	-	-	-	-	+
**M3 10^{8}**	-	+	-	-	+	-	+

Supplementary Figure S1. Presence and absence of clearance zones on Methylene blue agar plates by fungal isolates

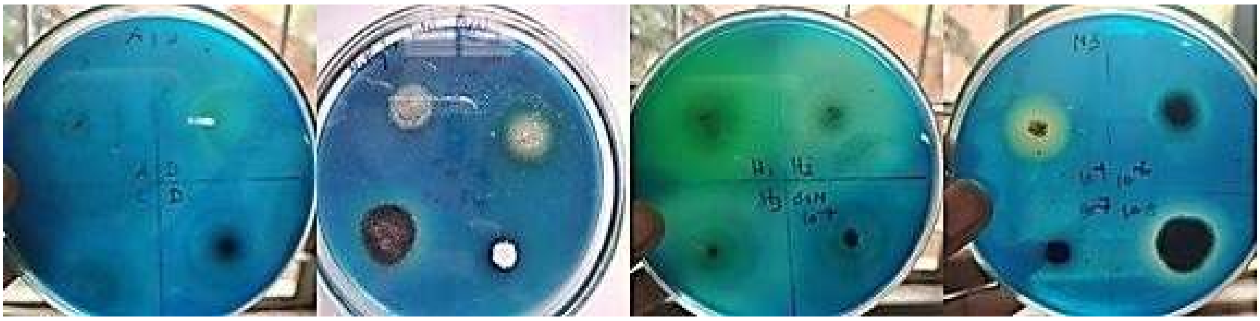

Supplementary Figure S2. Presence and absence of zone in Congo red plates by fungal isolates

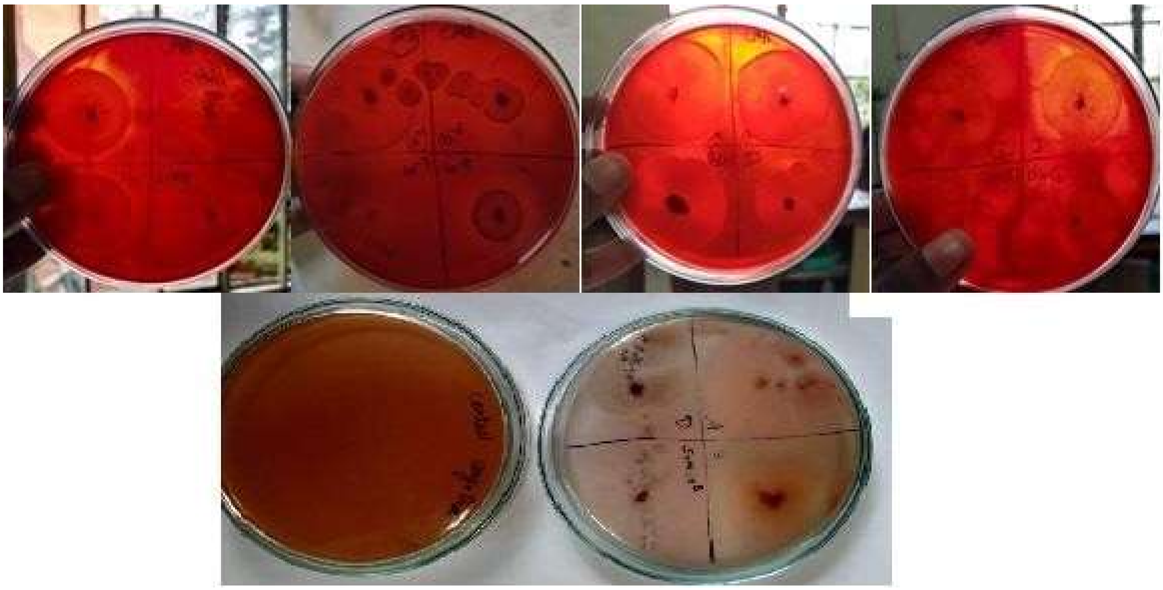

Supplementary Figure S3. Presence and absence of decolorization in Bromophenol blue plates by fungal isolates

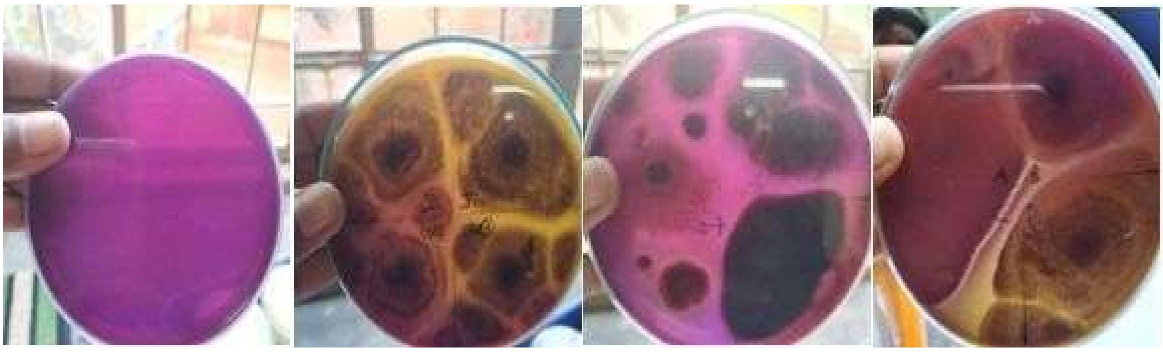

Supplementary Figure S4. Growth of fungal strains utilizing Tannic acid as sole carbon source in Mineral Salt Medium.

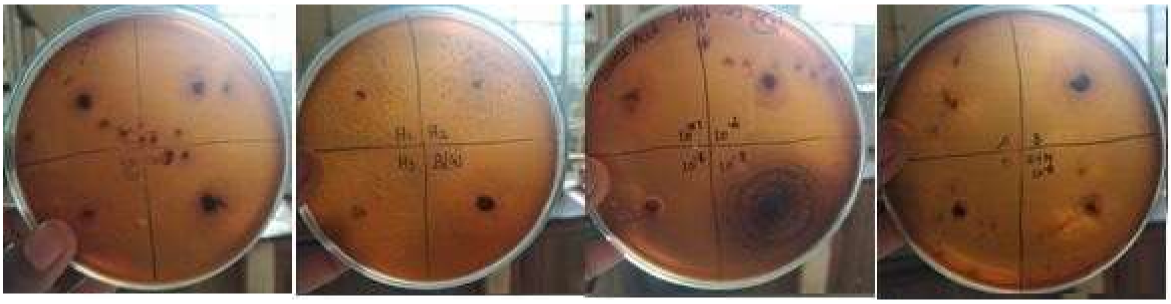

Supplementary Figure S5 Growth of fungal strains utilizing Guaiacol as sole carbon source in Mineral Salt Medium

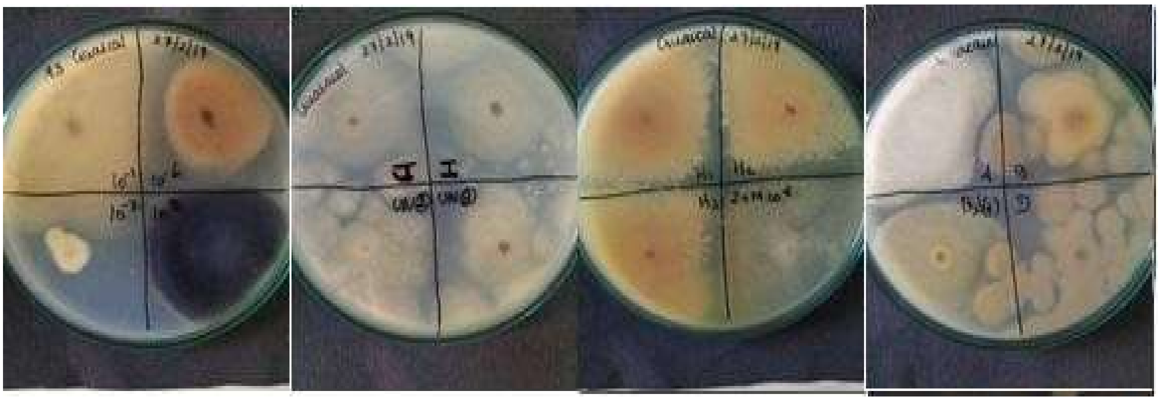

Supplementary Table S2 Colony morphology of fungal strains on PDA plates (obverse and reverse view) and microscopic view (40X)

Fungal strain	Colony Morphology on PDA plate		Spore arrangement	Tentative identity
	Obverse view	Reverse view		
A	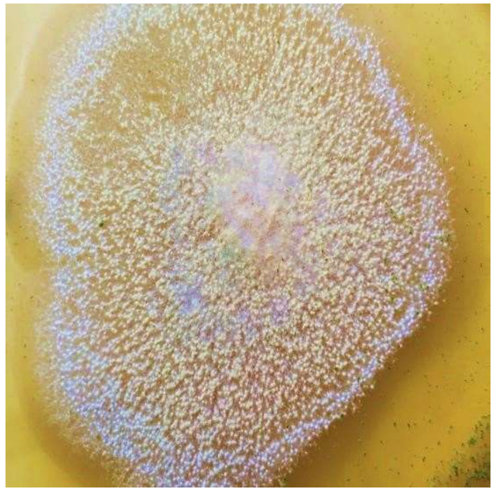	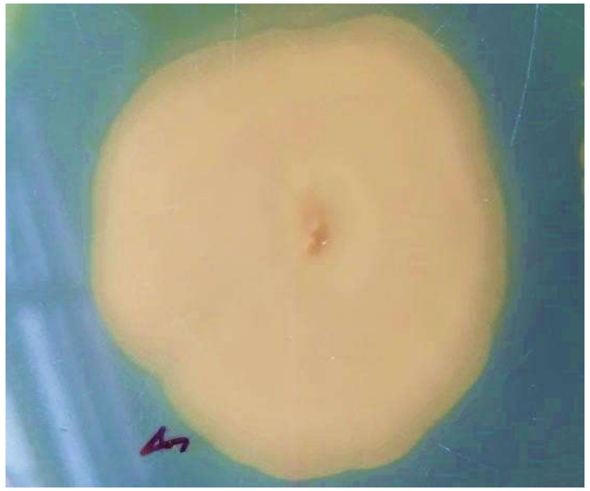	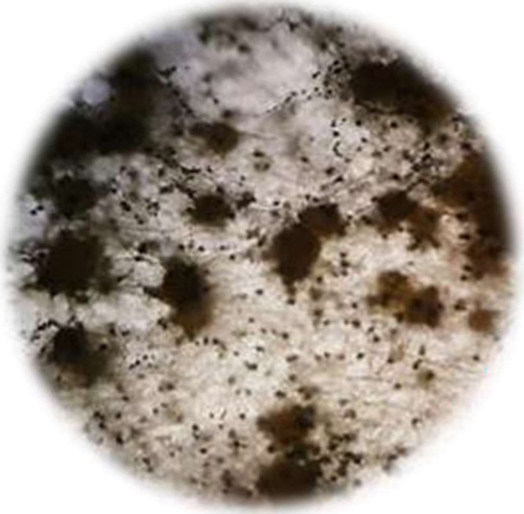	***Eurotium herbariorum (A. glaucus)***
B	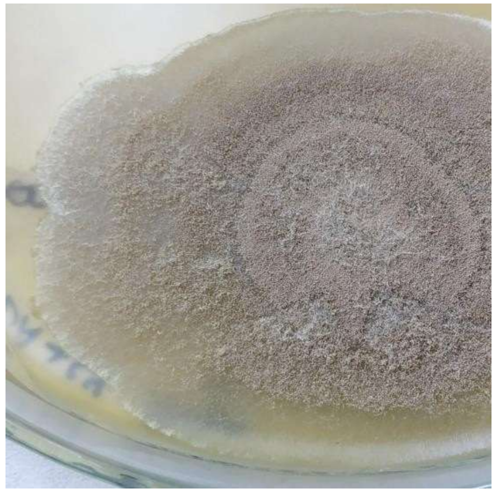	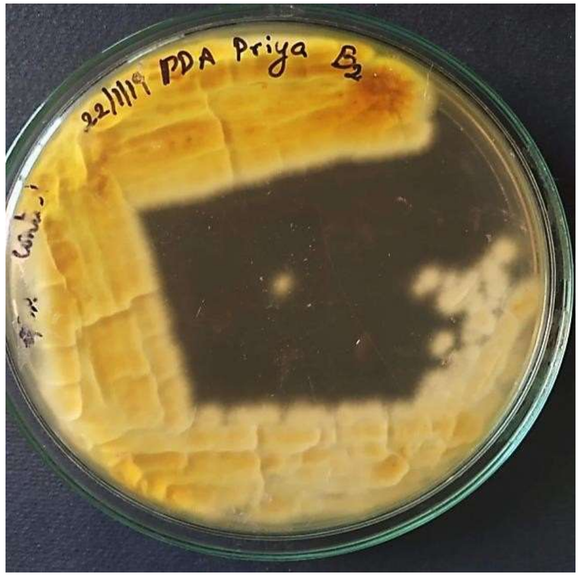	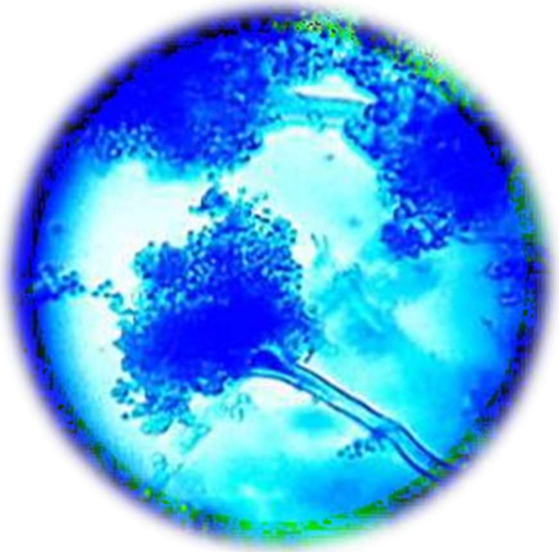	***Aspergillus terreus***

C	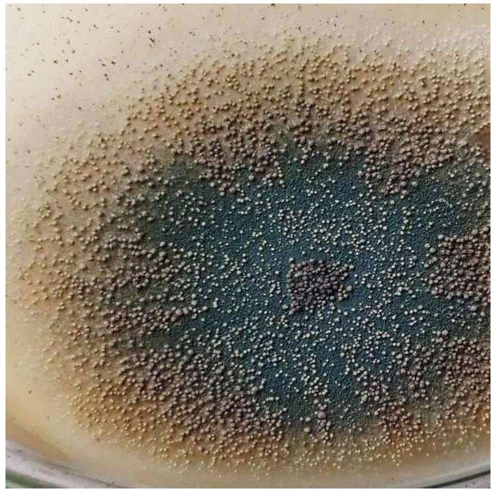	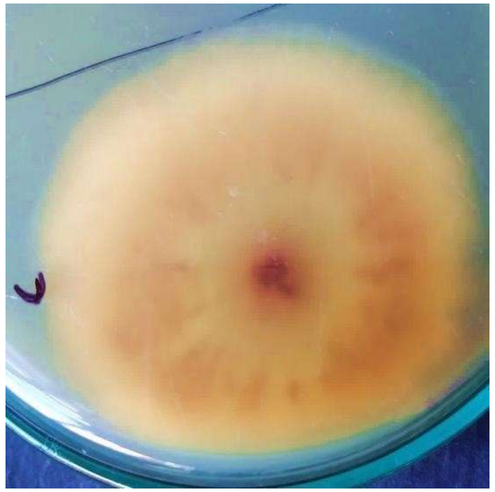	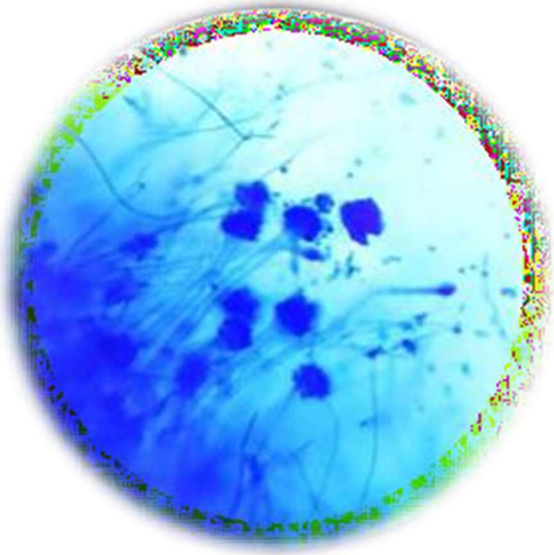	***Aspergillus fumigatus***
D2	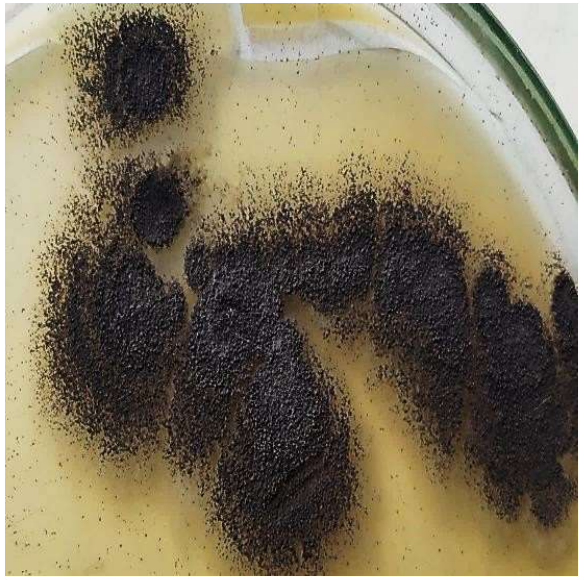	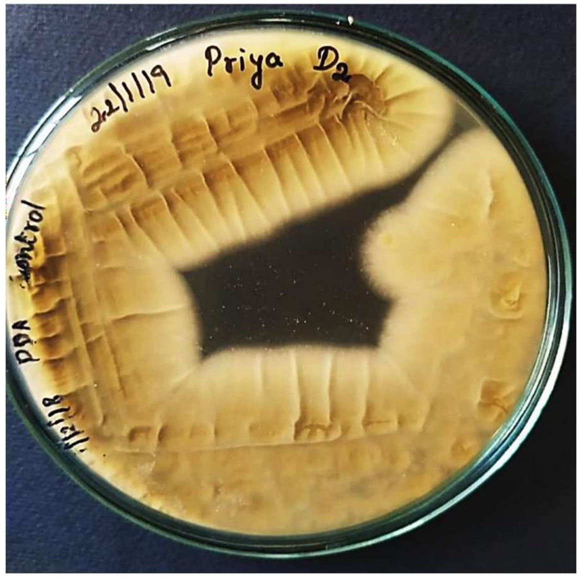	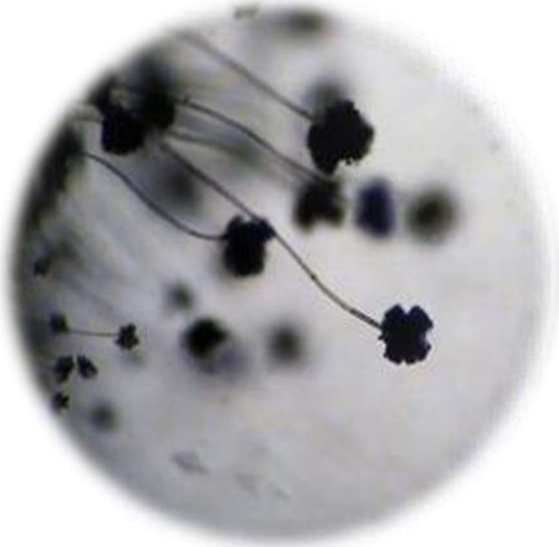	***Aspergillusniger***

H1	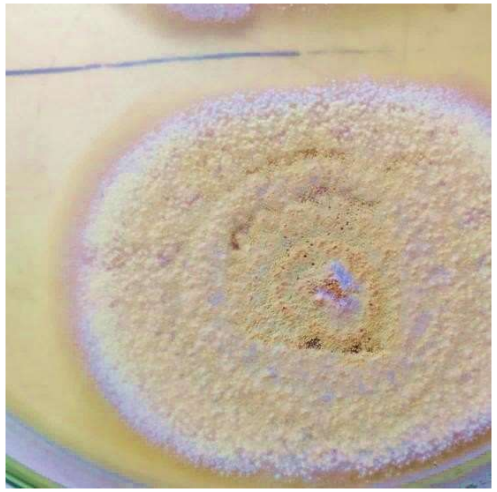	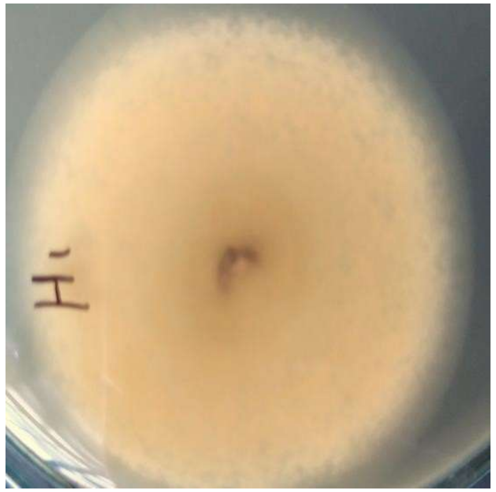	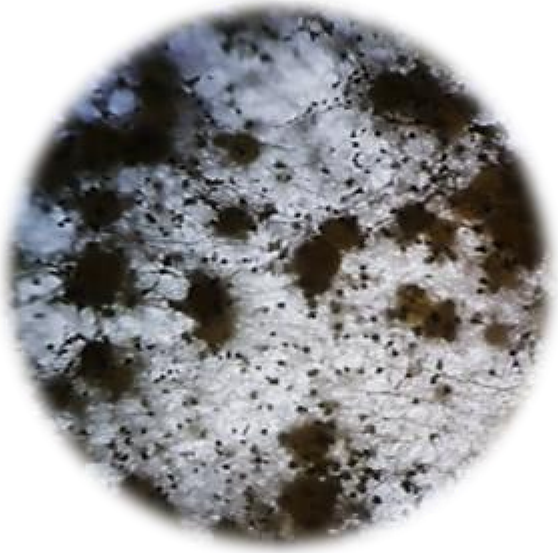	***Eurotium herbariorum (A.glaucus)***
H2	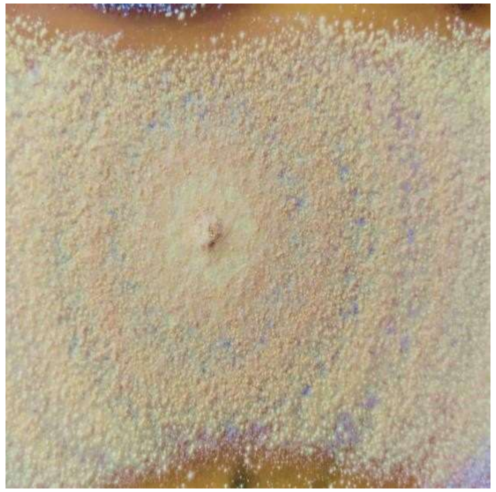	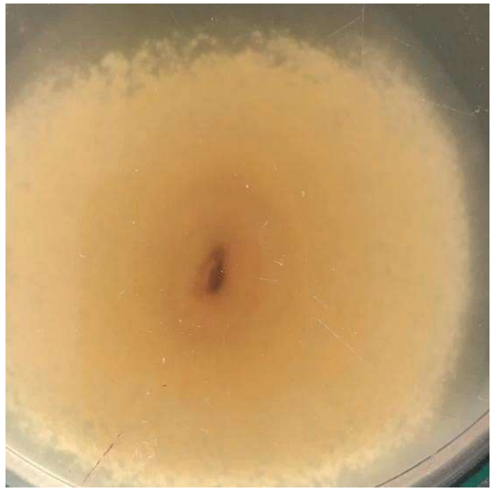	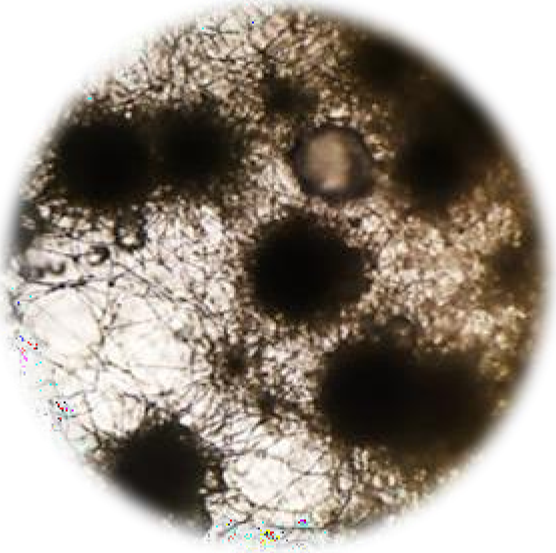	***Eurotium herbariorum (A.glaucus)***

H3	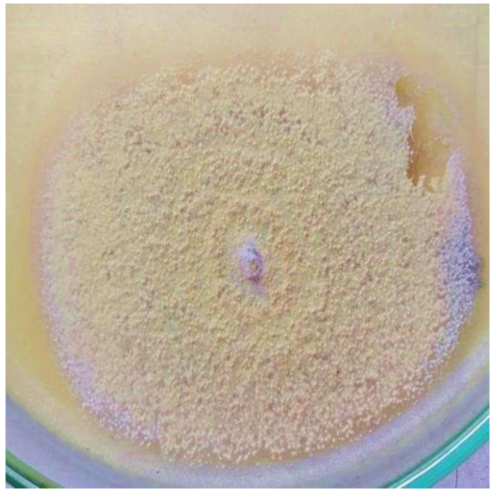	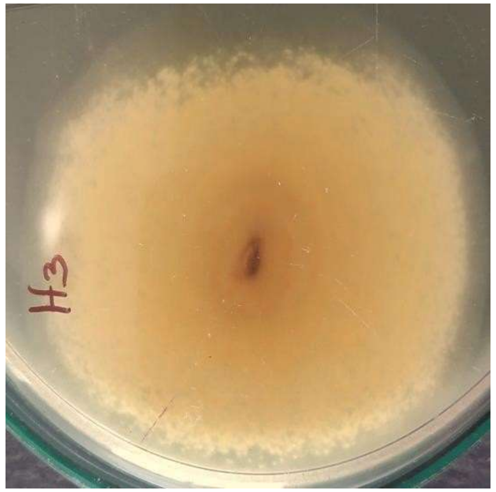	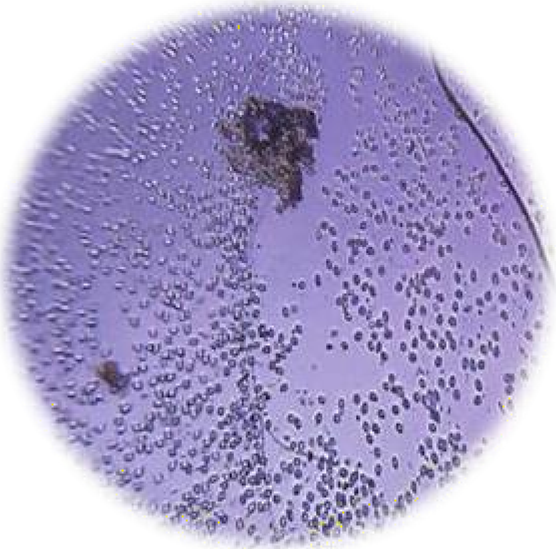	***Eurotium herbariorum (A.glaucus)*** **allomorph**
I	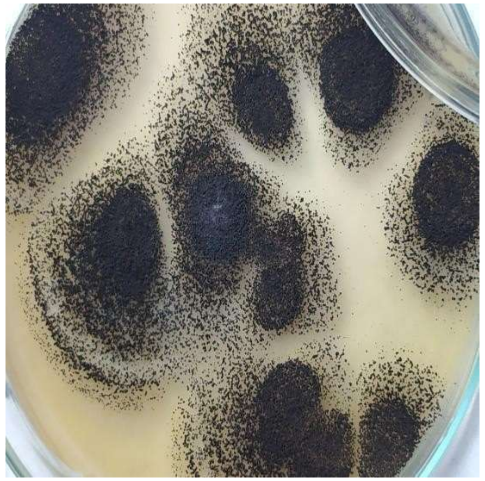	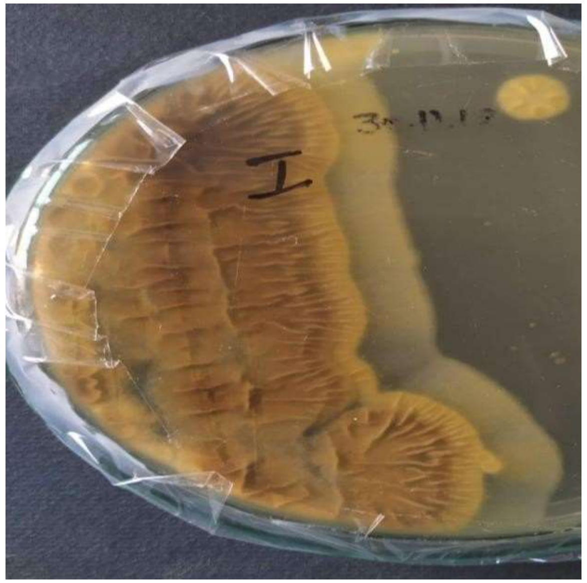	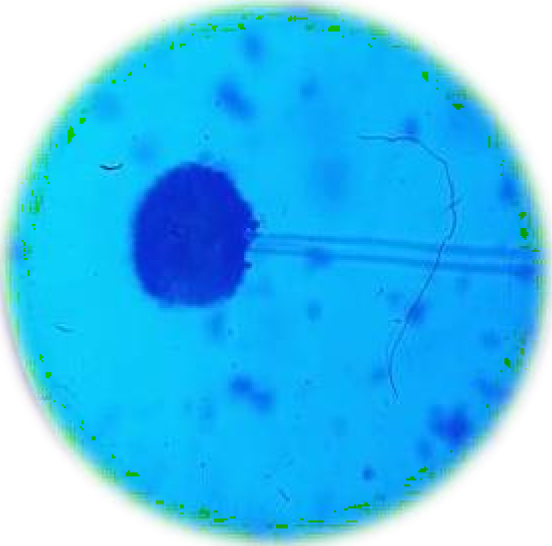	***Aspergillusniger***

J	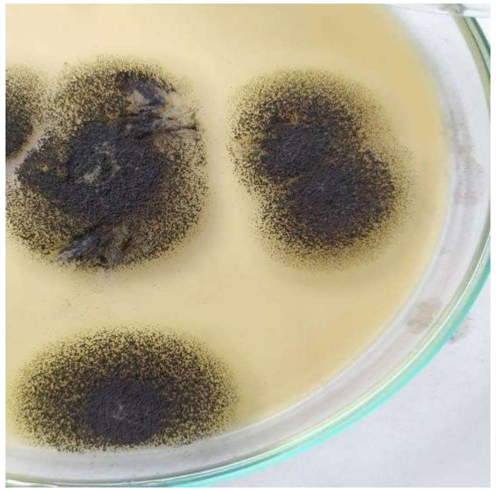	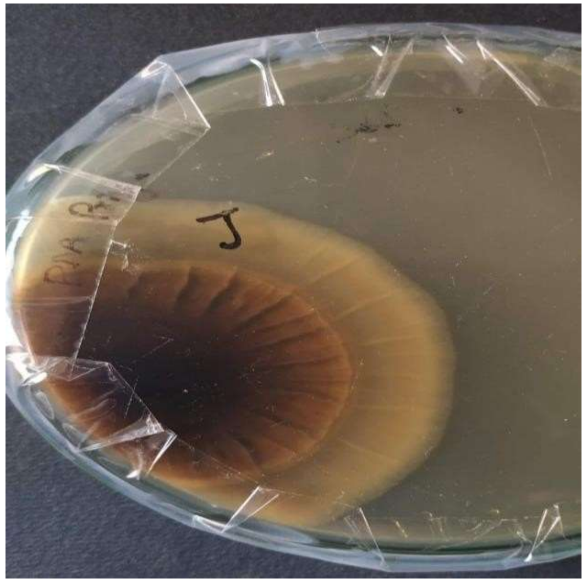	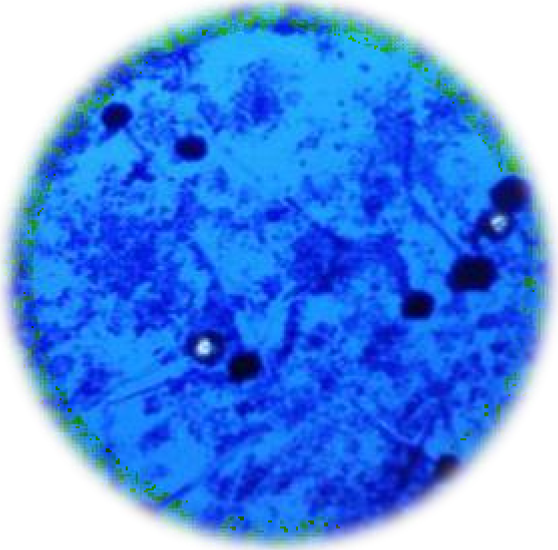	***Aspergillusniger***
M1	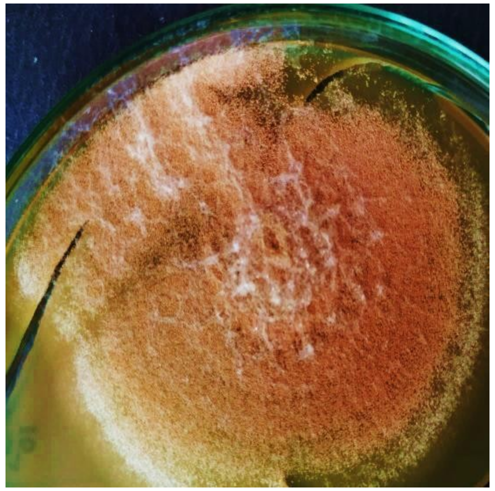	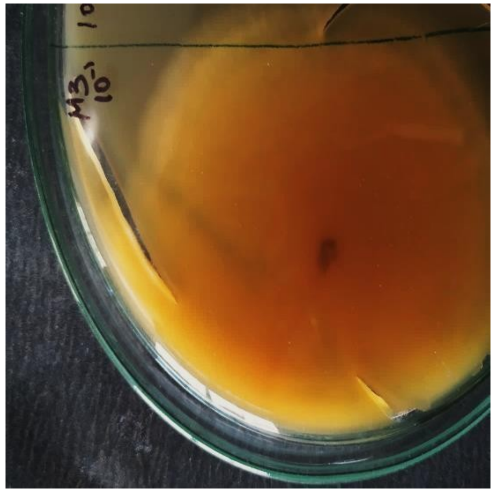	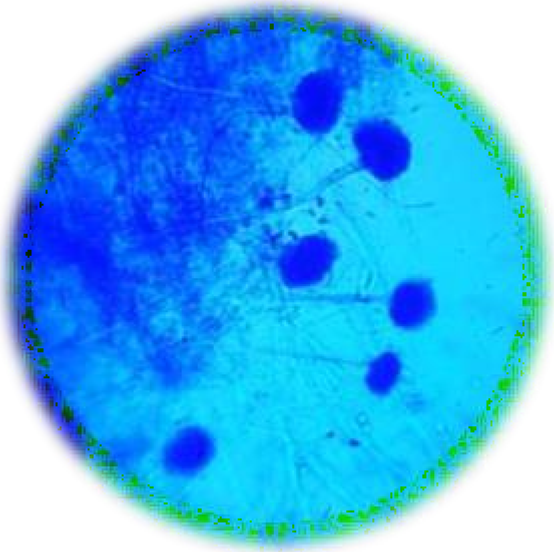	***Aspergillus terreus***

M6	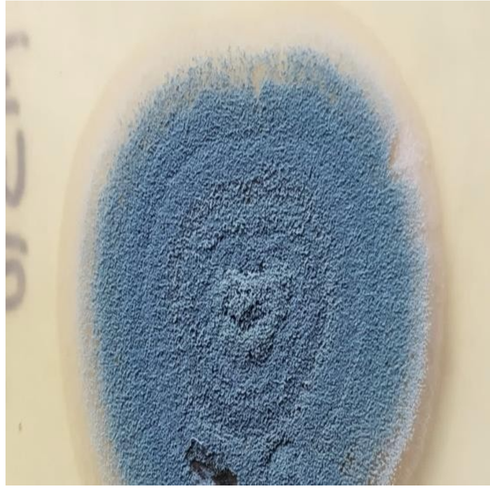	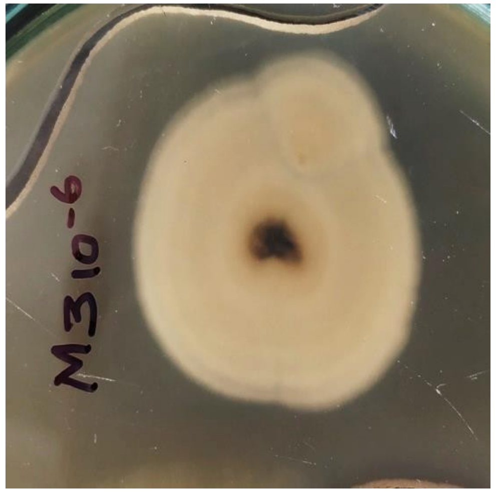	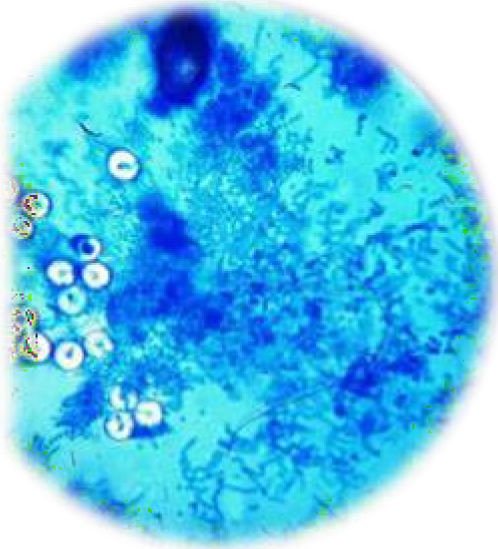	***Aspergillus (Emericella) nidulans***
M7	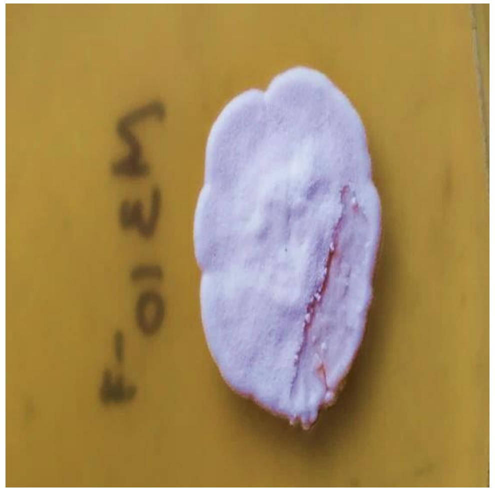	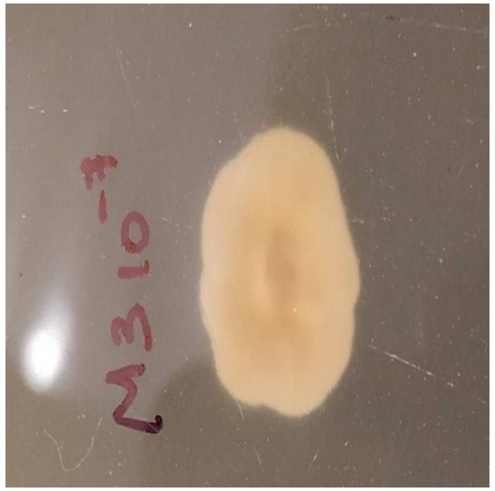	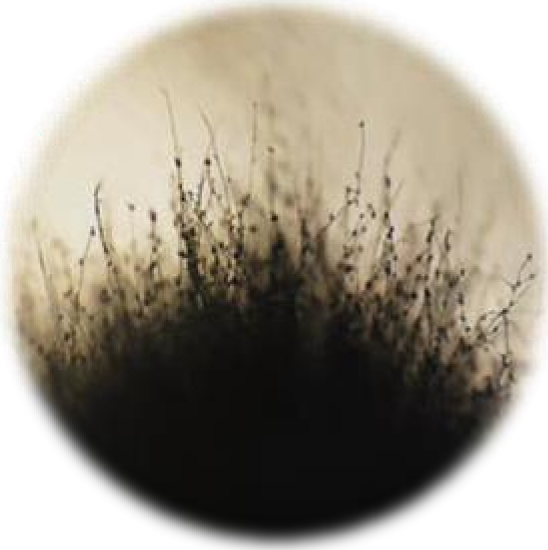	***Trichoderma viride***

M8	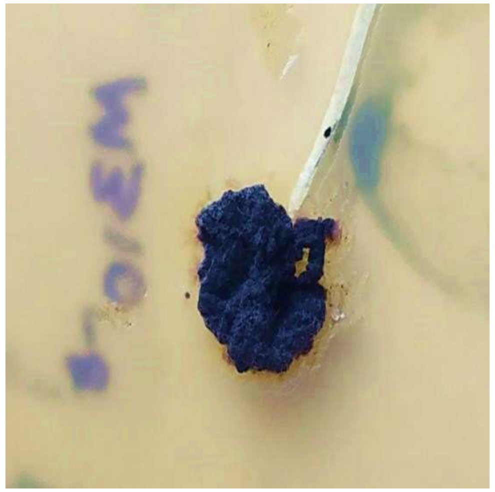	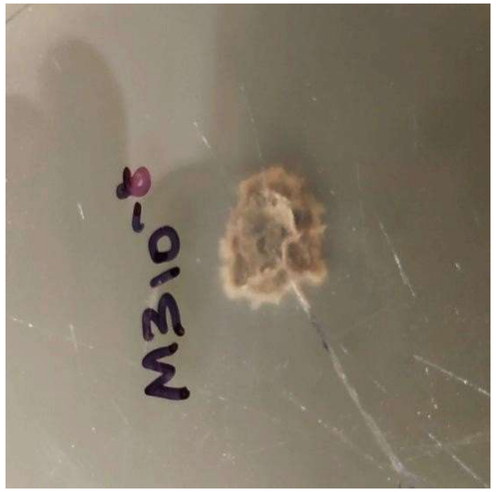	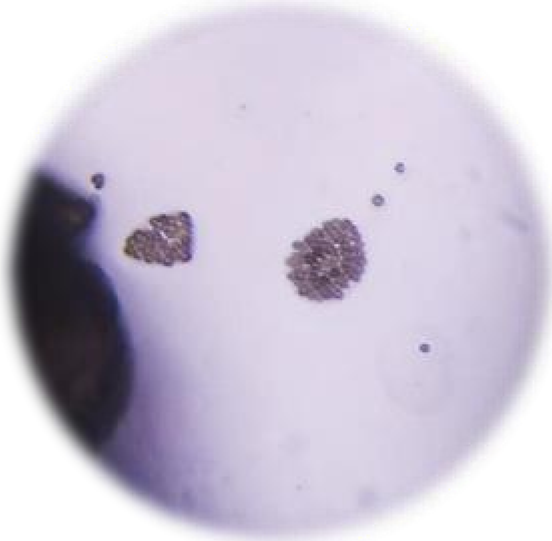	NA
SM108	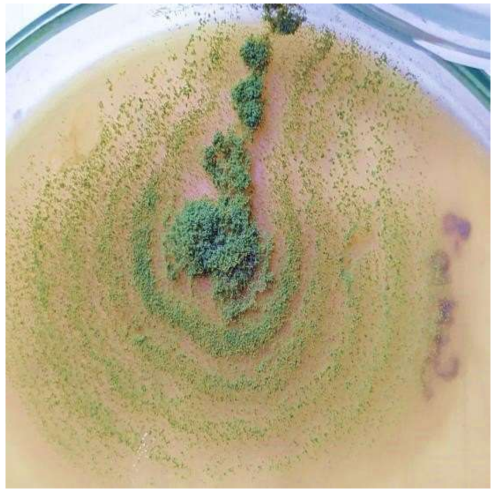	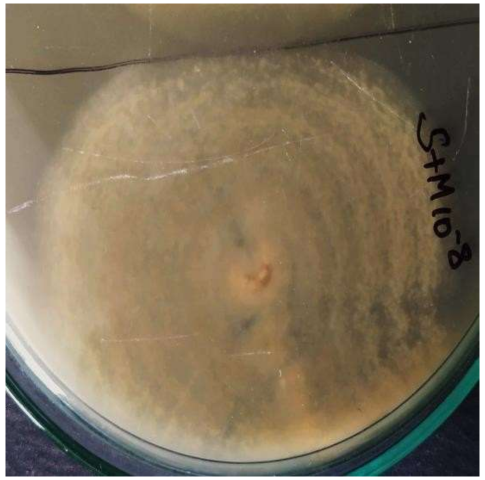	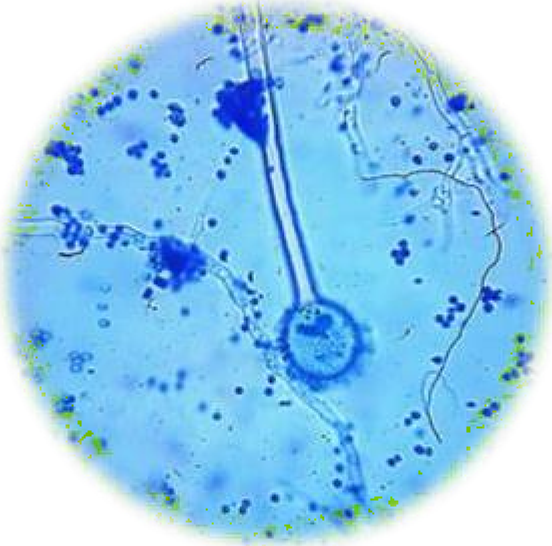	***Aspergillusflavus***

UN2	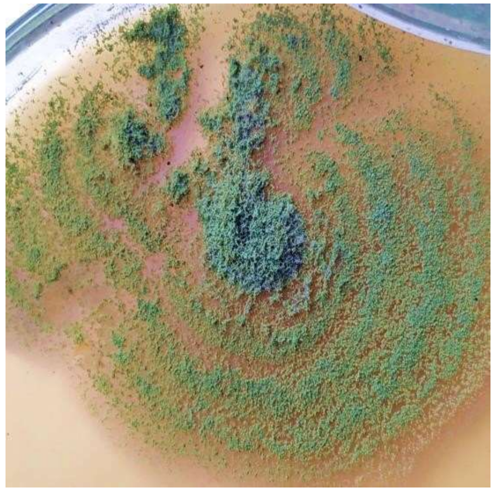	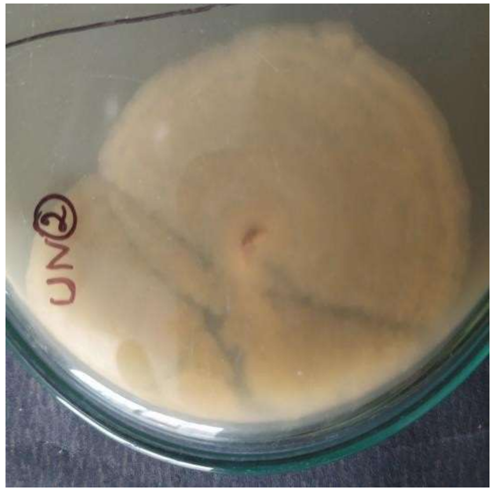	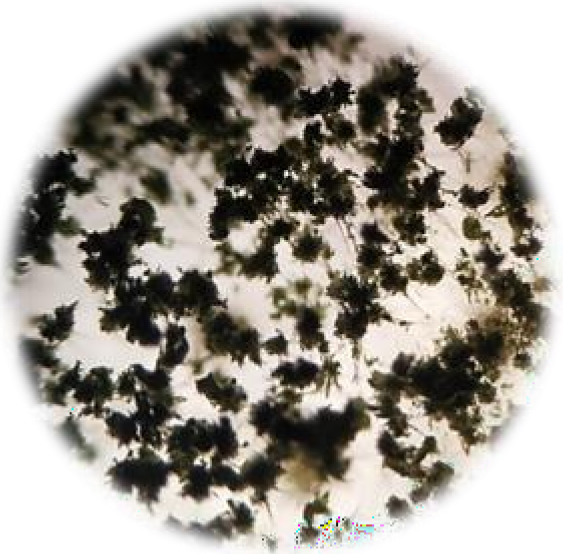	***Aspergillusflavus***
UN3	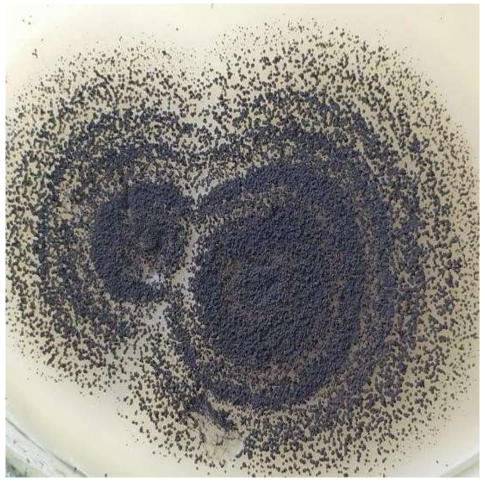	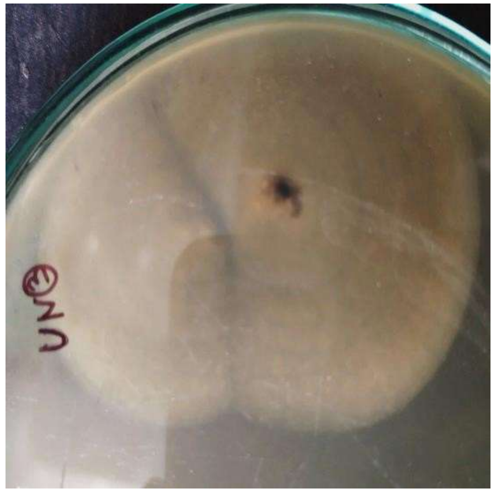	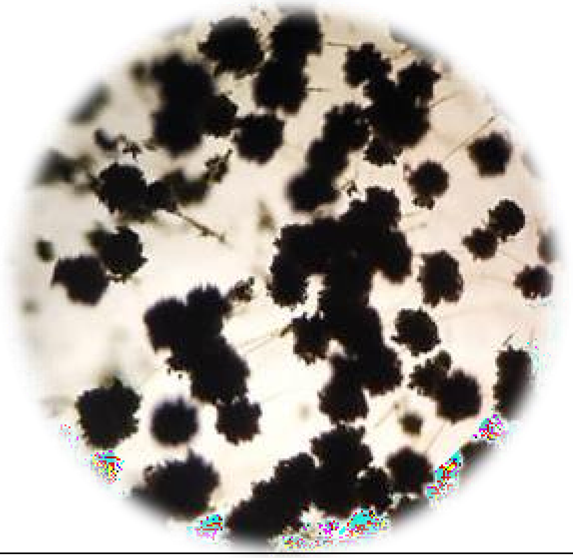	***Aspergillus niger***
